# Targeted/exome sequencing identified mutations in ten Chinese patients diagnosed with Noonan syndrome and related disorders

**DOI:** 10.1186/s12920-017-0298-6

**Published:** 2017-10-30

**Authors:** Shanshan Xu, Yanjie Fan, Yu Sun, Lili Wang, Xuefan Gu, Yongguo Yu

**Affiliations:** 1 Department of Pediatric Endocrinology/Genetics, Xin Hua Hospital affiliated to Shanghai Jiao Tong University School of Medicine, Shanghai Institute for Pediatric Research, 1665 Kongjiang Road, Shanghai, 200092 China; 2 Department of Pediatric, The First Affiliated Hospital of Xiamen University, Xiamen, Fujian 361003 China

**Keywords:** Noonan syndrome, Whole exome sequencing, PTPN11, RAF1, BRAF, Gene mutation

## Abstract

**Background:**

Noonan syndrome (NS) and Noonan syndrome with multiple lentigines (NSML) are autosomal dominant developmental disorders. NS and NSML are caused by abnormalities in genes that encode proteins related to the RAS-MAPK pathway, including PTPN11, RAF1, BRAF, and MAP2K. In this study, we diagnosed ten NS or NSML patients via targeted sequencing or whole exome sequencing (TS/WES).

**Methods:**

TS/WES was performed to identify mutations in ten Chinese patients who exhibited the following manifestations: potential facial dysmorphisms, short stature, congenital heart defects, and developmental delay. Sanger sequencing was used to confirm the suspected pathological variants in the patients and their family members.

**Results:**

TS/WES revealed three mutations in the *PTPN11* gene, three mutations in *RAF1* gene, and four mutations in *BRAF* gene in the NS and NSML patients who were previously diagnosed based on the abovementioned clinical features. All the identified mutations were determined to be de novo mutations. However, two patients who carried the same mutation in the *RAF1* gene presented different clinical features. One patient with multiple lentigines was diagnosed with NSML, while the other patient without lentigines was diagnosed with NS. In addition, a patient who carried a hotspot mutation in the *BRAF* gene was diagnosed with NS instead of cardiofaciocutaneous syndrome (CFCS).

**Conclusions:**

TS/WES has emerged as a useful tool for definitive diagnosis and accurate genetic counseling of atypical cases. In this study, we analyzed ten Chinese patients diagnosed with NS and related disorders and identified their corresponding*PTPN11, RAF1,* and *BRAF* mutations. Among the target genes, *BRAF* showed the same degree of correlation with NS incidence as that of *PTPN11* or *RAF1*.

## Background

A number of genetic syndromes are attributed to mutations in genes involved in the Ras/MAPK pathway (*PTPN11, SOS1, RAF1, KRAS, NRAS, BRAF, MAP2K1, SHOC2*, and *CBL*). These include Noonan syndrome (NS), Noonan syndrome with multiple lentigines (NSML), cardiofaciocutaneous syndrome (CFCS), and Costello syndrome (CS), which share overlapping clinical features and have been collectively termed RASopthies [1, 2].

NS is a rare disorder that was first defined by Dr. Jacqueline A. Noonan in 1963 [3]. NS is an autosomal dominant developmental disorder with an estimated prevalence of 1:1000 to 2500 births [4]. It is characterized by facial dysmorphisms, short stature, congenital heart defect, and variable degrees of developmental delay. Missense mutations in *PTPN11*, *SOS1*, *RAF1*, and *KRAS* account for approximately 50%, 10–13%, 3–17%, and <5% of all NS cases, respectively. De novo mutations account for 60% of all NS cases [5].

NSML was previously known as LEOPARD syndrome, which was derived from the primary symptoms that include multiple lentigines, electrocardiographic conduction defects, ocular hypertelorism, pulmonary stenosis, abnormal genitalia, growth retardation, and sensorineural deafness [6]. NSML is caused by carrying a heterozygous pathogenic variantin one of four specific genes, namely, *PTPN11*, *RAF1*, *BRAF*, and *MAP2K1*.

CFCS is characterized by cardiac abnormalities, specialcraniofacial appearance, and cutaneous abnormalities (eg, ichthyosis, eczema, pigmented moles and hemangiomas); Some researchers reported CFCS patients who also suffered acute lymphoblastic leukemia (ALL). Four genes are known to be led to CFCS syndrome, namely, *BRAF* (~75%), *MAP2K* and *MAP2K2* (~25%), and *KRAS* (<2%) [7, 8].

In the past, the standard genetic diagnostic process for NS was based on Sanger sequencing and single gene analysis for *PTPN11*. This can be followed by subsequent single-gene analyses for *SOS1*, *RAF1*, *KRAS*, *NRAS*, *BRAF*, and *MAP2K1* when no mutation was identified for *PTPN11*. This inefficient procedure was time-consuming and often led to additional economic burden for both the patients and clinicians. Recently, targeted/whole exome sequencing (TS/WES) has increasingly been employed for clinical diagnosis and has changed the paradigm of molecular diagnostic testing because of advantages, such as cost-effectiveness, generation of high-quality outputs, simplicity, and automated operation [9–11]. TS/WES is employed to obtain more comprehensive and gene-level information and generate a more accurate diagnosis. In particular, TS/WES is useful for clinicians when the phenotypes of sporadic patients are variable and complicated.

In the present study, we identified mutations in the *PTPN11*, *RAF1*, and *BRAF* genes using TS/WES in patients who had above-mentioned clinical features.

## Methods

### Subjects

By retrospectively reviewing the results generated from targeted sequencing/whole exome sequencing between 2014 and 2016, ten patients with mutations in genes involved in Noonan syndrome and related disorders were identified and presented in this report (six males, four females). The mean age was 3.8 years (range: 5 months to 10 years). All patients received physical examination, neurological/neuropsychiatric assessment, biochemical testing, echocardiography, karyotype analysis, and tandem mass test. Family history was routinely been recorded. Whole-genome copy number variation (CNV) array and enzyme activity tests related to mucopolysaccharidosis/mucolipidosisi were performed in some of the patients.

All patients enrolled in this study have signed informed consent by their parents, including allowing pictures, medical data been published.

### Whole exome sequencing

Peripheral blood samples were collected from the patients and their parentsafter informed consent was obtained. Genomic DNA (gDNA) was extracted using Lab-Aid Nucleic Acid (DNA) Isolation Kit (Zeesan, China) according to the manufacturer’s instructions.ClearSeq Inherited Disease or SureSelect Human All Exon V5 kit (Agilent, Santa Clara, CA, USA) were used for library preparation of targeted sequencing or whole exome sequencing, respectively. The resulting libraries were sequenced on a HiSeq 4000 platform (Illumina, San Diego, CA, USA) according to the manufacturer’s instructions for paired-end 150-bp reads. The minimal data amount was2.5Gb per sample for TS and 8Gb per sample for WES.Fastq-format reads were aligned to the human reference genome (GRCh37/hg19) using BWA-0.7.10 [12]. BAM files were manipulated using Picard tools-1.124. Base calling was performed following GATK best practice version 3 [13]. Quality metrics were evaluated - the average depth was 80× per sample, with at least 97% of the target region covered by 10× reads or more. The vcf files were then annotated using SnpEff version 4.2 [14]. Variants with >1% frequency in the population variant databases -1000Genomes Project, Exome Variant Server (EVS) and Exome Aggregation Consortium (ExAC) or > 5% frequency in the local database with 150 exome datasets were filtered, and subsequentlyintergenic, intronic, and synonymous variants were filtered, except those located at canonical splice sites. Candidate variants were then evaluated in the context of clinical presentation and inheritance mode. Selected variants were validated by Sanger sequencing in the proband and parents. Paternity was confirmed for de novo variants.

### Functional prediction of novel mutations

Unreported non-synonymous amino acid variants were predicted using MutationTaster (http://www.mutationtaster.org), SIFT (http://sift.jcvi.org), and PolyPhen-2 (http://genetics.bwh.harvard.edu/pph2/) to evaluate any potentially damaging effects. The potential changes in three-dimensional (3D) protein structure induced by the novel missense mutation were predicted using Swiss PDB viewer.

## Results

### Clinical presentations and comparison with literature

**Fig. 1:**
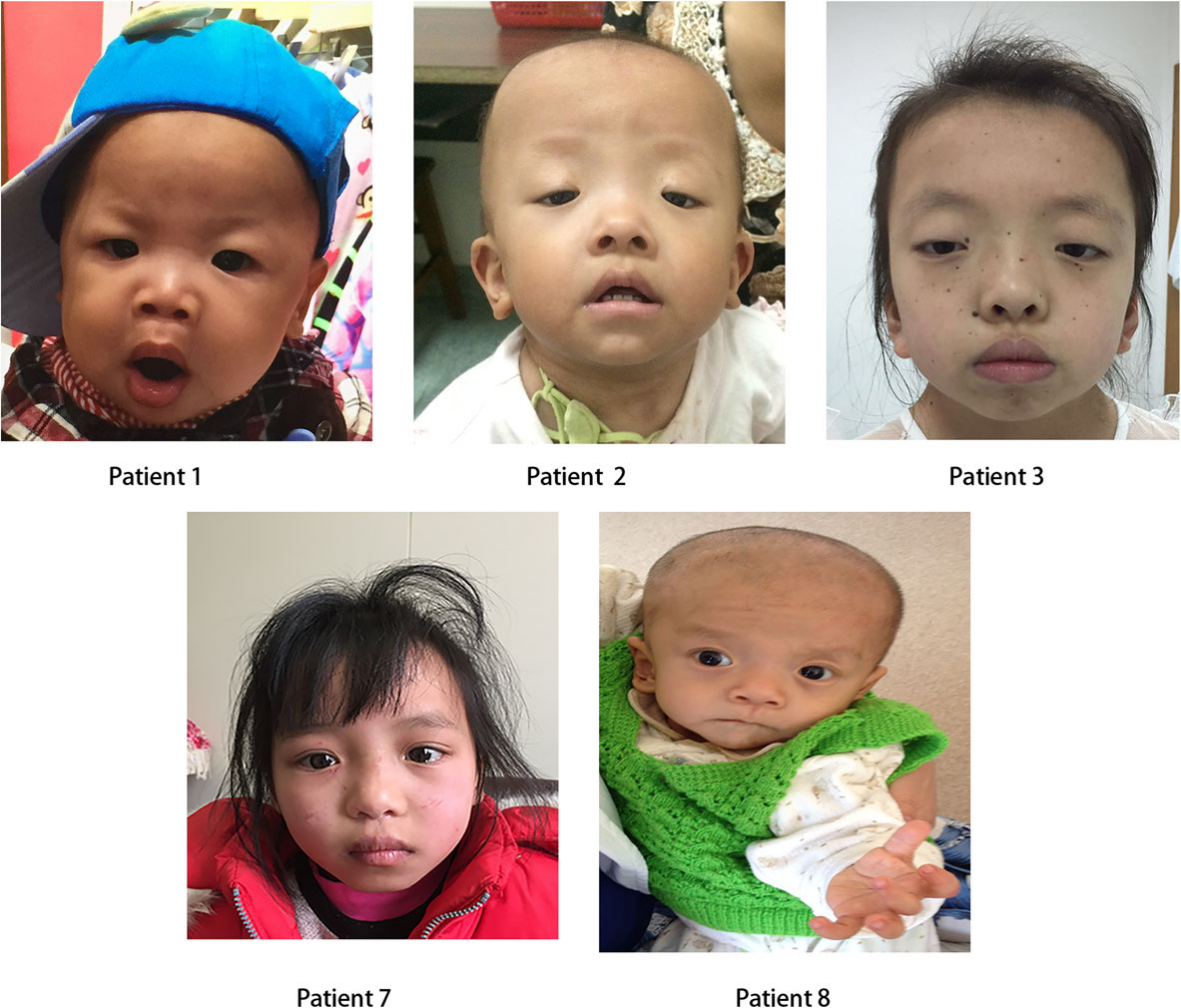
Potential facial dysmorphisms of cases in this study. Patients 1,2,7, and 8 were diagnosed with NS, while patient 3 was diagnosed with NSML

The detailed clinical features of the ten patients analyzed in our study are displayed in Table 1. Figure 1 shows the facial dysmorphisms of some of the patients(with consent obtained from parents for publication). All patients were sporadic cases.

**Table 1:** Clinical features of our patients

Patient	1	2	3	4	5	6	7	8	9	10
Sex	M	F	F	M	M	M	F	M	M	F
Age	1Y	15 M	10Y	11 M	10Y	5 M	9Y	10 M	4Y	14 M
Height(cm)	70	67	114.4	67	100.2	57	104.5	60.3	80	53
Karyotype	46,XY	46,XX	46,XX	46,XY	46,XY	46,XY	46,XX	46,XY	46,XY	46,XX
Congenital heart defect										
PVS	+	–	–	–	–	–	+	–	–	–
ASD	+	+	–	+	–	–	–	+	+	–
PDA	–	–	–	–	–	–	–	–		–
VSD	–	–	–	–	–	–	–	–	+	–
Mitral or tricuspid valve defects	–	+	–	–	–	–	–	–	–	–
Hypertrophic cardiomyopathy (HCM)	–	–	+	–	–	–	–	–	–	–
Short stature (<3rd centile)	–	+	+	–	+	–	+	+	+	+
Short webbed neck	–	–	+	–	–	–	–	–	–	–
Chest deformity	–	–	+	–	–	+	–	+	–	–
Characteristic facies										
Low-set posteriorly rotated ears with fleshy helices	+	+	+	+	+	+	+	+	+	+
Downslanting palpebral fissures	+	+	+	–	–	+	–	+	+	–
Palpebral ptosis	–	+	+	–	–	+	–	+	+	–
Wide-spaced eyes	–	+	+	+	+	+	+	+	+	–
Epicanthal folds	–	–		–	–		–	–	–	–
Deeply grooved philtrum	–	+	–	–	–	+	+	+	–	–
High wide peaks of the vermilion	–	+	+	–	–	+	+	+	–	–
Low posterior hairline	–	–	–	–	+		+	–	–	–
Thick curly hair or thin sparse hair	–	–	+	–	–	+	+	–	–	+
Micrognathia		+	–	–	–	+	–	+	–	+
Macrocephaly	–	–	–	–	–	–	+	–	–	–
Malocclusion	–	–	+	–	–	–	–	–	–	–
Excess nuchal skin	+	–	–	–	–	–	+	–	–	–
Others										
Developmental delay or cognitive deficit	+	+	–	+	+	+	+	+	+	+
Lymphatic dysplasias	–	–	–	–	–	–	–	–	–	–
Feeding difficulties	+	+	–	–	–	+	–	+	–	+
Renal anomaly	–	–	–	–	–	–	–	–	–	–
Increased bleeding tendency	–	–	–	–	–	–	–	–	–	–

*ASD* atrial septal defect, *VSD* ventricular septal defect, *HCM* hypertrophic cardiomyopathy, *PDA* patent ductus arteriosus, *PVS* pulmonary valve stenosis

+present, −not present

All studied individuals exhibited dysmorphic facial features, mild-to-moderate cognitive deficits, short stature, feeding difficulties, skeletal anomalies, and hypotonia. The most common facial features could be found in NS patients including prominent forehead, downslanting palpebral fissures, ptosis, thick palpebral lids, epicanthal folds, flat nasal bridge, and low-set helical ears. Seven out of ten (70%) patients had short stature (<3 centile). Atrial septal defect (ASD) was the most common cardiac defect (5/10, 50%), followed by pulmonary valve stenosis (PVS) (2/10, 20%). Hypertrophic cardiomyopathy (HCM) and multiple lentigines were observed in patient 3, who was diagnosed with NSML.

### Identification of disease-causing mutations

As shown in Table 2, TS/WES identified three genes harboring a total of ten mutations in the ten patients after filtering and manual review of the genes according to clinical presentation. The genes that carried mutations were *PTPN11* (3/10 = 30%), *RAF1* (3/10 = 30%), and *BRAF* (4/10 = 40%). In this study, *BRAF* was found to be the most common pathological gene in the NS patients, followed by *PTPN11* and *RAF1*. In our study, all detected mutations were de novo mutations and not present in their parents, with paternity confirmed.

**Table 2:** Mutations identified by WES in ten patients

Patient	Phenotype	Gene	Refseq	Nuclei acid change	Amino acid change	Allele state	Chromosomal position(hg19)	GnomAD frequency	Accession Number
1	NS	PTPN11	NM_002834.3	c.923A > G	A308S	het	Chr12:112,915,524	0	rs121918455
2	NS	RAF1	NM_002880.3	c.770C > T	S257 L	het	Chr3:12,645,699	0	rs80338796
3	NSML	RAF1	NM_002880.3	c.770C > T	S257 L	het	Chr3:12,645,699	0	rs80338796
4	NS	RAF1	NM_002880.3	C.781C > A	P261T	het	Chr3:12,645,688	0	rs121434594
5	NS	PTPN11	NM_002834.3	c.236A > G	Q79R	het	Chr12:112,888,220	0	rs121918466
6	NS	BRAF	NM_004333.4	c.1403 T > C	F468S	het	Chr7:140,481,405	4.062e-6	rs397507473
7	NS	PTPN11	NM_002834.3	c.209A > G	K70R	het	Chr12:112,888,193	0	rs397516801
8	NS	BRAF	NM_004333.4	c.770A > G	Q257R	het	Chr7:140,501,302	0	rs180177035
9	NS	BRAF	NM_004333.4	c.1403 T > G	F468C	het	Chr7:14,081,405	0	Not reported
10	NS	BRAF	NM_004333.4	c.1785 T > G	F595 L	het	Chr7:140,453,150	0	rs121913341

*NS* Noonan syndrome, *NSML* Noonan syndrome with multiple lentigines

Patient 3, who presented multiple lentigines and carried a NSML-associated *RAF1* mutation (c.770C > T, p.S257 L), was diagnosed with NSML [15–18], whereas patient 2, who carried the same mutation but lacked multiple lentigines, was diagnosed with NS (Fig. 1). The diagnosis of patient 2 contradicted the previous claim that the S257 L mutation is always linked to hypertrophic cardiomyopathy.

### Functional prediction of the novel mutant protein

**Fig. 2:**
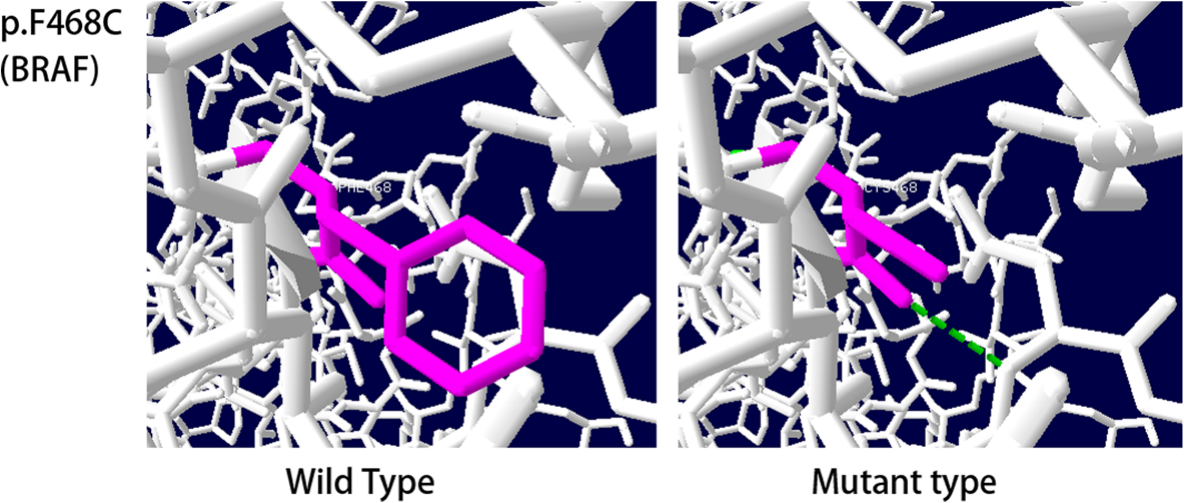
3D structural models of BRAF containing the mutant sites

We identified onenovel mutation in *BRAF* (c.1403 T > G, p.F468C) genes in patients 9. This variant has not been previously reported in the Human Gene Mutation Database, the 1000 Genomes Database, orGnomAD database at the time of writing of this manuscript. It was predicted to be “probably damaging” with a score of 0.996 for c.1403 T > G, p.F468C based on the PolyPhen-2 software, predicted to “affect protein function” with a score of 0.00 by the SIFT software, and classified as “disease-causing” by the Mutation Taster software. Change in the 3D protein structure induced by these novel missense mutation was predicted using Swiss PDB Viewer. The wild-type and mutant BRAF protein 3D structural model are illustrated in Fig. 2. Thewild-type residue was located in highly conserved domains. In BRAF, residue 468 is located in CR3, a highly conserved region that encodes a part of the kinase activity domain. The F468C mutation generates a smaller residue and potentially causes the loss of external interactions.

## Discussion

In this study, we verified the prevalence of *PTPN11*, *RAF1*, and *BRAF* mutations in Chinese patients diagnosed with NS and related disorders via TS/WES. We identified a total of ten mutations in the ten patients. All patients who carried *PTPN11* and *BRAF* mutations were diagnosed with NS. Two patients who carried the same *RAF1* mutation presented different features and were separately diagnosed with NS and NSML.

*PTPN11* is thought to be the most common pathogenic gene that causes NS, followed by *RAF1*. *BRAF* mutations are very rarely found in NS cases [1, 15]. *PTPN11* encodes a key protein, a member of the protein tyrosine phosphatase (PTP) family, whichresponds to growth factors, hormones, and cell adhesion molecules [19]. RAF1 is a downstream factor of RAS signaling in the MAPK pathway that encodes a protein with 648 amino acids and comprises three domains, namely, CR1, CR2, and CR3. NS and NSML are both associated with mutations in *PTPN11* and *RAF1*. However, some of the mutations potentially drive the NS phenotype, while other mutations are predicted to produce the NSML phenotype [20].

The prevalent*PTPN11* mutations Y279C, A308S, and T468 M account for 65% of total NS cases and produce loss-of-function SHP2 domain mutants that lack catalytic activity [15]. A study of genotype-phenotype correlation reported that NS patients harboring *PTPN11* mutations, especially a codon 308 mutation, had higher incidence of pulmonic stenosis than NS patients without *PTPN11* mutations [15, 21]. Compared with other patients harboring the codon 308 mutation reported in previous literature, patient 1 has pulmonary valve stenosis (vs 36/51,70.6%) and short stature (vs 39/51, 76.5%) but does not present pectus deformities (vs 39/50,78%) nor cryptorchidism (26/31, 83.9%) [15]. Thus, these findings confirmed different clinical presentations of *PTPN11* mutations.

*PTPN 11*K70R has not been published in the literature, but in Clinvar, itis classified as “Likely Pathogenic”.This variant has been identified in 5 affected individuals and segregates with symptoms of Noonan syndrome in one family. As lack of clinical data from other study, we cannot compare the phenotype among the patients who had the same K70R mutation.

The RAF1 mutations 770C > T (p. S257 L) and 781C > A (p. P261T) detected in this study were both clustered in the CR2 domain, which is important for regulatory phosphorylation and binding with the 14–3-3 protein. In a previous study, *RAF1* was thought to be associated with HCM because all patients that carried the S257 L mutation were diagnosed with HCM, and two of them died from severe HCM [18]. This genotype-phenotype correlation appeared to be domain-specific, since the region encoding the 14–3-3 consensus site was affected in the HCM patients. In our study, both patients 2 and 3 carried the S257 L mutation, which was associated with both NS and NSML [18]. Patient 3 displayed typical HCM echocardiography and multiple lentigines in the face, so an NSML diagnosis should be considered. However, patient 2 presented normal interventricular septum(IVS) and mildly thickened left ventricular posterior wall (LVPW), so an HCM diagnosis cannot be confirmed at this point. The patient did not present lentigines, so he was diagnosed with NS.

Sarkozy reported a female whose early clinical presentationwas typical of NS but eventually developed hearing loss and lentigines, which are typical phenotypesof NSML, as the disease progressed [7]. Lentigines usually appear at an early age(eg, 4–5 years old), and increase until puberty. Similarly, the penetrance of left ventricular hypertrophy (LVH) is also age-dependent. The LVH of HCM often becomes apparent during adolescence or young adulthood. Patient 2 was only 15 months old upon admission and can thus develop LVH later in life. Therefore, patient 2 requires further follow-up to determine whether a novel phenotype will emerge.

The *BRAF* gene is thought to be the primary cause of CFCS. *BRAF* mutations account for around 50%–75% of all CFCS cases, but is implicated in only a small fraction of NS and NSML cases (<2%) [7, 22–24]. Sarkozy and Koudova identified some individuals who were clinically diagnosed with NS or NSML that carried *BRAF* mutations [21, 25]. However, NS- or NSML-related *BRAF* mutations aren’t as same as those that occur in CFCS, suggesting a genotype-phenotype correlation. Unfortunately, the mechanisms underlying this phenomenon remain to be elucidated. The c.770A > G(p.Q257R) mutation is the most widespread CFCS pathogenic variant [8] and was also detected in patient 8. Assuming a genotype-phenotype correlation, patient 8 should present features of CFCS. However, he had characteristic facies, cardiac defects, short stature, abnormal brain MRI, failure to thrive, and relative developmental delay, but lacked typical cutaneous abnormalities and musculoskeletal and ocular abnormalities; hence, he was diagnosed with NS instead of CFCS. This specific case expanded the mutational spectrum of the *BRAF* gene in NS and highlighted the genetic heterogeneity of *BRAF*.

We detected two mutations at residue 468 in the *BRAF* gene. Patient 6 carried a c.1403 T > C (p.F468S) mutation, which has been reported in a previous study [26]. Patient 9 carried a c.1403 T > G (p.F468C) mutation affecting the same protein. However, F468Cwas never been reported in NS or related disorderspreviously.Interestingly, itwas detected in paraffin-embedded tumoursepecimens of a hairy cell leukemia (HCL) patient [27] and a colorectal cancer patient [28].There is evidence from in vitro and in vivotransfection experiments [29] that F468C mutation leads to increased activity of BRAF and may thus be disease-defining mutation of HCL or colorectal cancer. By sequencing BRAF genefrom normal gastric biopsies of the HCL patient, germline mutation is excluded [27].Our report is the first time to detect F468C germline mutation in a non-cancer patient.Patients 6&9presented similar clinical characteristics, which supported the idea that the phenotype resulting from *BRAF* mutations is allele-specific and suggested that residue 468 may be a “hotspot” mutation site in Chinese patients.

The ten patients in this study shared features, such as congenital heart defect, short stature, and special facies, that led to difficulties in defining CFCS, NSML, or NS using clinical criteria. Next-generation sequencing (NGS) is a rapid and economical technique that provides molecular-based diagnosis for clinically overlapping conditions. NGS facilitates early disease diagnosis, especially for patients with mild/moderate, atypical features, and can potentially direct clinicians towards more reliable genetic counseling and clinical treatment of the patients.

## Conclusions

Overall, we verified the prevalence of *PTPN11*, *RAF1*, and *BRAF* mutations in NS and related disorders in the Chinese population. *BRAF* showed the same degree of correlation with NS incidence as that of *PTPN11* or *RAF1*. The same mutation can result in different phenotypes, suggesting that the phenotypes arising from *RAF1* or *BRAF* defects are likely to be allele-specific.

## Abbreviations

ALL: Acute lymphoblastic leukemia
ASD: Atrial septal defect
CFCS: Cardiofaciocutaneous syndrome
CNV: Whole-genome copy number variation
CS: Costello syndrome
gDNA: Genomic DNA
HCL: Hairy cell leukemia
HCM: Hypertrophic cardiomyopathy
IVS: Interventricular septum
LVH: Left ventricular hypertrophy
LVPW: Left ventricular posterior wall
NGS: Next-generation sequencing
NS: Noonan syndrome
NSML: Noonan syndrome with multiple lentigines
PVS: Pulmonary valve stenosis
TS/WES: Targeted sequencing/whole exome sequencing

## References

[1] Tartaglia M, Gelb BD, Zenker M (2011). Noonan syndrome and clinically related disorders. Best Pract Res ClinEndocrinolMetab.

[2] Myers A, Bernstein JA, Brennan ML, Curry C, Esplin ED, Fisher J, Homeyer M, Manning MA, Muller EA, Niemi AK, Seaver LH, Hintz SR, Hudgins L (2014). Perinatal features of the RASopathies: Noonan syndrome, cardiofaciocutaneous syndrome and Costello syndrome. Am J Med Genet A.

[3] Noonan JA (1968). Hypertelorism with turner phenotype. A new syndrome with associated congenital heart disease. Am J Dis Child.

[4] Cole RB (1980). Noonan syndrome a historical perspective. Pediatrics.

[5] Shaw AC, Kalidas K, Crosby AH, Jeffery S, Patton MA (2007). The natural history of Noonan syndrome: a long-term follow-up study. Arch Dis Child.

[6] Sarkozy A, Digilio MC, Dallapiccola B (2008). Leopard syndrome. Orphanet J Rare Dis.

[7] Sarkozy A, Carta C, Moretti S, Zampino G, Digilio MC, Pantaleoni F, Scioletti AP, Esposito G, Cordeddu V, Lepri F, Petrangeli V, Dentici ML, Mancini GM, Selicorni A, Rossi C, Mazzanti L, Marino B, Ferrero GB, Silengo MC, Memo L, Stanzial F, Faravelli F, Stuppia L, Puxeddu E, Gelb BD, Dallapiccola B, Tartaglia M, Germline BRAF (2009). Mutations in Noonan, LEOPARD, and cardiofaciocutaneous syndromes: moleculardiversity and associated phenotypic spectrum. Hum Mutat.

[8] Niihori T, Aoki Y, Narumi Y, Neri G, Cave H, Verloes A (2006). Germline KRAS and BRAF mutations in cardio-facio-cutaneous syndrome. Nat Genet.

[9] Dixon-Salazar TJ, Silhavy JL, Udpa N, Schroth J, Bielas S, Olvera J, Bafna V, Zaki MS, Abdel-Salam GH, Mansour LA, Selim L, Abdel-Hadi S, Marzouki N, Ben-Omran T, Al-Saana NA, Sonmez FM, Celep F, Azam M, Hill KJ, Collazo A, Fenstermaker AG, Novarino G, Akizu N, Garimella KV, Sougnez C, Russ C, Gabriel SB, Gleeson JG (2012). Exome sequencing can improve diagnosis and alter patient management. SciTransl Med.

[10] Lepri FR, Scavell R, Digilio MC, Gnazzo M, Grotta S, Dentici ML, Pisaneschi E, Sirleto P, Capolino R, Baban A, Russo S, Franchin T, Angioni A, Dallapiccola B (2014). Diagnosis of Noonan syndrome and related disorders using target next generation sequencing. BMC Medical Genetics.

[11] Coromilas A, Wynn J, Haverfield E, Chung WK (2015). Nonspecific phenotype of Noonan syndrome diagnosed by whole exome sequencing. Clinical Case Reports.

[12] Li H, Durbin R (2009). Fast and accurate short read alignment with burrows-wheeler transform. Bioinformatics.

[13] McKenna A, Hanna M, Banks E, Sivachenko A, Cibulskis K, Kernytsky A, Garimella K, Altshuler D, Gabriel S, Daly M, DePristo MA (2010). The genome analysis toolkit: a MapReduce framework for analyzing next-generation DNA sequencing data. Genome Res.

[14] Cingolani P, Platts A, Wang le L, Coon M, Nguyen T, Wang L, Land SJ, Lu X, Ruden DM (2012). A program for annotating and predicting the effects of single nucleotide polymorphisms, SnpEff: SNPs in the genome of Drosophila Melanogaster strain w1118; iso-2; iso-3. Fly (Austin).

[15] Tartaglia M, Kalidas K, Shaw A, Song X, Musat DL, van der Burgt I, Brunner HG, Bertola DR, Crosby A, Ion A, Kucherlapati RS, Jeffery S, Patton MA, Gelb BD (2002). PTPN11 Mutations in Noonan Syndrome: molecular Spectrum, genotype-phenotype correlation, and phenotypic heterogeneity. Am J Hum Genet.

[16] Sarkozy A, Conti E, Seripa D, Digilio MC, Grifone N, Tandoi C, Fazio VM, di Ciommo V, Marino B, Pizzuti A, Dallapiccola B (2003). Correlation between PTPN11 gene mutations and congenital heart defects in Noonan and LEOPARD syndromes. J Med Genet.

[17] Razzaque MA, Nishizawa T, Komoike Y, Yagi H, Furutani M, Amo R, Kamisago M, Momma K, Katayama H, Nakagawa M, Fujiwara Y, Matsushima M, Mizuno K, Tokuyama M, Hirota H, Muneuchi J, Higashinakagawa T, Matsuoka R (2007). Germline gain-of-function mutations in RAF1 cause Noonan syndrome. Nat Genet.

[18] Pandit B, Sarkozy A, Pennacchio LA, Carta C, Oishi K, Martinelli S, Pogna EA, Schackwitz W, Ustaszewska A, Landstrom A, Bos JM, Ommen SR, Esposito G, Lepri F, Faul C, Mundel P, JP LS, Tenconi R, Selicorni A, Rossi C, Mazzanti L, Torrente I, Marino B, Digilio MC, Zampino G, Ackerman MJ, Dallapiccola B, Tartaglia M, Gelb BD (2007). Gain-of-function RAF1 mutations cause Noonan and LEOPARD syndromes with hypertrophic cardiomyopathy. Nat Genet.

[19] Neel BG, Gu H, Pao L (2003). The 'Shp'ing news: SH2 domain-containing tyrosine phosphatases in cell signaling. Trends Biochem Sci.

[20] Sarkozy A, Obregon MG, Conti E, Esposito G, Mingarelli R, Pizzuti A, Dallapiccola BA (2004). Novel PTPN11 gene mutation bridges Noonan syndrome, multiple lentigines/LEOPARD syndrome and Noonan-like/multiple giant cell lesion syndrome. Eur J Hum Genet.

[21] Hopper RK, Feinstein JA, Manning MA, Benitz W, Hudgins L (2015). Neonatal pulmonary arterial hypertension and Noonan syndrome: two fatal cases with a specific RAF1 mutation. Am J Med Genet A.

[22] Nyström AM, Ekvall S, Berglund E, Björkqvist M, Braathen G (2008). DuchenK, Noonan and cardio-facio-cutaneous syndromes: two clinically and genetically overlapping disorders. J Med Genet.

[23] Nava C, Hanna N, Michot C, Pereira S, Pouvreau N, Niihori T, Aoki Y, Matsubara Y, Arveiler B, Lacombe D, Pasmant E, Parfait B, Baumann C, Héron D, Sigaudy S, Toutain A, Rio M, Goldenberg A, Leheup B, Verloes A, Cavé HCFC (2007). Noonan syndromes due to mutations in RAS/MAPK signaling pathway: genotype/phenotype relationships and overlap with Costello syndrome. J Med Genet.

[24] Schulz AL, Albrecht B, Arici C, van der Burgt I, Buske A, Gillessen-Kaesbach G, Heller R, Horn D, Hübner CA, Korenke GC, König R, Kress W, Krüger G, Meinecke P, Mücke J, Plecko B, Rossier E, Schinzel A, Schulze A, Seemanova E, Seidel H, Spranger S, Tuysuz B, Uhrig S, Wieczorek D, Kutsche K, Zenker M (2008). Mutation and phenotypic spectrum in patients with Cardio-Facio-Cutaneous and Costello syndrome. Clin Genet.

[25] Koudova M, Seemanova E, Zenker M (2009). Novel BRAF mutation in a patient with LEOPARD syndrome and normal intelligence. Eur J Med Genet.

[26] Rodriguez-Viciana P, Tetsu O, Tidyman WE, Estep AL, Conger BA, Cruz MS, McCormick F, Rauen KA (2006). Germline mutations in genes within the MAPK pathway cause cardio-facio-cutaneous syndrome. Science.

[27] Tschernitz S, Flossbach L, Bonengel M, Roth S, Rosenwald A, Geissinger E, Alternative BRAF (2014). Mutations in BRAF V600E-negative hairy cellleukaemias. Br J Haematol.

[28] Yuen ST, Davies H, Chan TL, Ho JW, Bignell GR, Cox C, Stephens P, Edkins S, Tsui WW, Chan AS, Futreal PA, Stratton MR, Wooster R, Leung SY (2002). Similarity of the phenotypic patterns associated with BRAF and KRAS mutations in colorectal neoplasia. Cancer Res.

[29] Ikenoue T, Hikiba Y, Kanai F, Aragaki J, Tanaka Y, Imamura J, Imamura T, Ohta M, Ijichi H, Tateishi K, Kawakami T, Matsumura M, Kawabe T, Omata M (2004). Different effects of point mutations within the B-Raf glycine-rich loop in colorectal tumors on mitogen-activated protein/extracellular signal-regulated kinase kinase/extracellular signal-regulated kinase and nuclear factor kappaB pathway and cellular transformation. Cancer Res.

